# Genetic Characterization of ExPEC-Like Virulence Plasmids among a Subset of NMEC

**DOI:** 10.1371/journal.pone.0147757

**Published:** 2016-01-22

**Authors:** Bryon A. Nicholson, Aaron C. West, Paul Mangiamele, Nicolle Barbieri, Yvonne Wannemuehler, Lisa K. Nolan, Catherine M. Logue, Ganwu Li

**Affiliations:** 1 Department of Veterinary Microbiology and Preventive Medicine, College of Veterinary Medicine, Iowa State University, Ames, Iowa, United States of America; 2 Department of Chemistry, College of Liberal Arts and Sciences, Iowa State University, Ames, Iowa, United States of America; 3 Department of Veterinary Diagnostic and Production Animal Medicine, College of Veterinary Medicine, 1802 University Blvd, Iowa State University, Ames, Iowa, 50011, United States of America; 4 Department of Veterinary Preventive Medicine, College of Veterinary Medicine, Nanjing Agricultural University, Nanjing, Jiangsu, 210095, P. R. China; University of Münster, GERMANY

## Abstract

Neonatal Meningitis *Escherichia coli* (NMEC) is one of the most common causes of neonatal bacterial meningitis in the US and elsewhere resulting in mortality or neurologic deficits in survivors. Large plasmids have been shown experimentally to increase the virulence of NMEC in the rat model of neonatal meningitis. Here, 9 ExPEC-like plasmids were isolated from NMEC and sequenced to identify the core and accessory plasmid genes of ExPEC-like virulence plasmids in NMEC and create an expanded plasmid phylogeny. Results showed sequenced virulence plasmids carry a strongly conserved core of genes with predicted functions in five distinct categories including: virulence, metabolism, plasmid stability, mobile elements, and unknown genes. The major functions of virulence-associated and plasmid core genes serve to increase *in vivo* fitness by adding multiple iron uptake systems to the genetic repertoire to facilitate NMEC’s survival in the host’s low iron environment, and systems to enhance bacterial resistance to host innate immunity. Phylogenetic analysis based on these core plasmid genes showed that at least two lineages of ExPEC-like plasmids could be discerned. Further, virulence plasmids from Avian Pathogenic *E*. *coli* and NMEC plasmids could not be differentiated based solely on the genes of the core plasmid genome.

## Importance

Neonatal meningitis associated *Escherichia coli* (NMEC) as well as some other types of extra-intestinal pathogenic E. coli (ExPEC) are largely defined by the large plasmid; however relatively few ExPEC-like plasmids in NMEC have been sequenced and relatively few studies have examined their effects on pathogenicity. In this study we sequenced 9 large ExPEC-like plasmids from cases of neonatal bacterial meningitis to determine the core set of genes, and to determine if an evolutionary relationship between ExPEC plasmids of NMEC and other ExPEC exists. Our results show the core plasmid genome of NMEC strains to include genes related to virulence and metabolism as well as a high degree of relatedness to other large ExPEC plasmids.

## Introduction

*Escherichia coli* is one of the world’s most well studied organisms. Ubiquitous in nature and prevalent in biotechnology, it is also one of the most commonly isolated pathogens from human disease. Though some strains are specialized to cause disease inside the intestinal tract, others are equipped to cause extraintestinal disease [1–3]. These extraintestinal pathogenic *E*. *coli* (ExPEC) include neonatal meningitis *E*. *coli* (NMEC), one of the predominant agents of neonatal bacterial meningitis [4]. Neonatal bacterial meningitis has a mortality rate between 15 to 40% [5] with 30% of survivors showing serious neurological sequelae including profound intellectual disability, epilepsy, or deafness [4]. Reviews suggest that NMEC are acquired by neonates from their mothers perinatally [5, 6]. From the initial site of colonization, NMEC transcytose the gastrointestinal tract into the bloodstream, and from there, they traverse the blood brain barrier (BBB) (the tight barrier formed by human brain microvascular endothelial cells (HBMEC)) into the central nervous system (CNS). Since successful CNS invasion requires a high bacteremia, NMEC must survive in the bloodstream, a trait that is mediated by two bacterial components, an anti-phagocytic capsule and outer membrane protein A (OmpA), which has an anti-complement role. In addition, NMEC’s replication in immune cells may play a role in achieving the requisite bacteremia. Also, NMEC’s traversal of HBMEC is preceded by its attachment to the BBB via Type 1 pili and OmpA, with invasion itself being mediated by Ibe proteins, FimH (tip adhesin of Type 1 pili), OmpA, and Cytotoxic Necrotizing Factor 1 (CNF1). K1 capsule may also contribute to invasion by preventing lysosomal fusion, allowing delivery of live bacteria into the CNS [5, 6].

Plasmids also seem to contribute to NMEC virulence. A recent study by Kariyawasam and colleagues indicates that a large plasmid increases the virulence of NMEC RS218 *in vivo* [7], while previous studies have shown that the acquisition of plasmids is one of the primary sources of genetic variance and virulence genes in different *E*. *coli* pathotypes [8]. The majority of these plasmids has been identified as IncF type plasmids [9], encoding pathogenicity islands, metabolic genes, antimicrobial resistance genes, and hypothetical genes. Acquisition of these plasmids may provide an advantage for survival during infection and in suboptimal environments, as evidenced by multiple studies showing that these plasmids confer a fitness advantage to NMEC during in host tissues [7, 10, 11]. Further, plasmids are associated with an increase in NMEC virulence in animal models [7, 10, 11], and cause cross-species and cross pathogenic group gain of virulence function [12].

Here, we seek to better understand the evolution of large ExPEC-like virulence plasmids in NMEC and ascertain their similarity to plasmids of other ExPEC pathotypes. While plasmids from NMEC strains S88 (O45:K1), S286 (O78:K1), RS218 (O18:K1) and CE10 (O7:K1) have been sequenced and are available in the public domain, it is our contention that these plasmids likely do not account for the full diversity of ExPEC-like virulence plasmid sequences in NMEC. We base this hypothesis on the variability found among NMEC in their possession of plasmid-associated genes [13]. We further contend that this knowledge gap could inhibit future study to identify rational strategies designed to control NMEC-caused disease. We will address some of these shortcomings by sequencing a collection of ExPEC-like virulence plasmids in NMEC strains, identify orthologous genes shared between these large ExPEC-like plasmids in an effort to identify their core and accessory plasmid genomes, ascertain the phylogenetic relationships between NMEC plasmids and determine their similarities to plasmids occurring in other ExPEC.

## Materials and Methods

### Bacterial Strains

A total of 11 *E*. *coli* isolates from cases of human neonatal meningitis were used as a source of plasmids in this study (Table 1). These isolates are part of a larger collection, containing 91 neonatal meningitis *E*. *coli* (NMEC) isolated from the Netherlands and United States. The bacterial strains were serogrouped at the *E*. *coli* Reference Center (Pennsylvania State University), genotyped for chromosomal and plasmid virulence genes and plasmid replicon types, and assigned to phylogenetic groups as previously described [13–15].

**Table 1:** Strains used in this study. Serogroups, phylogroups and genome statistics of selected sequenced NMEC strains used in the study.

Plasmid	Phylogroup	O:K	Cluster [1]	Size (bp)	GC%	ORFs	Source
**pNM14**	C	7:80	2	126,885	49.4	136	This Study
**pNM15**	B2	18:1	8	148,173	51.3	157	This Study
**pNM16**	B2	83:1	8	130,692	49.3	136	This Study
**pNM19**	B2	-:1	8	133,585	48.8	142	This Study
**pNM26**	B2	21:-	6	152,078	49.4	168	This Study
**pNM36**	B2	14:1	7	129,262	49.2	131	This Study
**pNM38**	B2	18:1	8	128,645	49.7	136	This Study
**pNM49**	B2	auto:1	8	121,081	49.6	119	This Study
**pNM58**	B2	18ac:1	8	157,260	49.7	157	This Study
**pS88**	B2	45:1	6	133,853	49.3	157	NC_011747.1
**pS286**	C	78:-	-	97,818	48.6	104	HF922624.1
**pAPEC-O1-ColBM**	B2	1:1	-	174,241	49.6	199	DQ381420.1
**pAPEC-O2-ColV**	B2	2:1	-	184,501	49.2	203	AY545598.5
**pAPEC-O103-ColBM**	B1	103:	-	124,705	50.8	126	NC_011964
**p1ColV5155**	B2	2:1	-	194,170	49.4	199	CP005930
**pVM01**	-	-:28	-	151,002	49.5	150	NC_010409
**pAPEC-1**	B1	78:-	-	103,275	45.1	164	CP000836
**pAPEC-O78-ColV**	-	78:-	-	144,859	50.4	158	NZ_CP010316

### Virulence Gene Amplification

As described in prior work, PCR was performed to determine the presence of chromosomal and plasmid derived virulence genes [13, 16]. Briefly, all strains were removed from freezer stock and struck to MacConkey agar plates. Isolates were then transferred to LB broth and grown at 37°C overnight. Bacterial DNA was harvested using the boil preparation method previously described [17]. For PCR analysis, isolates were tested for 205 genes associated with virulence and their allelic variants, antimicrobial resistance, plasmid replicons, and pathogenicity islands [13]. Primers were obtained from Integrated DNA Technologies (Coralville, Iowa). Replicon typing was carried out using the methodology described by Johnson [8] using multiplex PCR. Phylogrouping was carried out using the revised method of Clermont, assigning strains to 8 phylogenetic groups [18].

### Selection of strains for sequencing

Strains for sequencing were selected based initially on the presence or absence of nine known plasmid virulence genes (*ompTp*, *hlyF*, *cvaC*, *etsA*, *iutA*, *iroN*, *tsh*, *iss*, *and sitC*) reported in previous studies as indicative of virulence plasmids and which may play a role in pathogenesis [9, 19]. To be considered virulence plasmids in this analysis, plasmids must contain at least two known plasmid virulence genes. Candidate plasmid containing strains were further refined by the number of virulence genes detected by PCR. Plasmids containing variable patterns of the previous nine aforementioned virulence genes were selected for sequencing. When presented with multiple strains possessing the same pattern of virulence genes, strains originating from different serogroups were selected followed by, phylogenetic groups, and capsule types in order to increase likelihood of sequencing genetically distinct plasmids.

### Plasmid Isolation

The selected strains were grown overnight in 5ml of Luria Bertani (LB) broth, the bacterial cells pelleted by centrifugation, and their plasmid DNA isolated using Qiagen midi plasmid prep kits according to manufacturer’s specifications (Venlo, Netherlands). The plasmid DNA pellet was re-suspended in Qiagen buffer EB. Plasmid DNA concentration was measured using a NanoDrop 1000 (Thermo Scientific, Wilmington, DE).

Plasmid separation was achieved using pulse field gel electrophoresis (PFGE) as described by Tivendale et al (6). PFGE was carried out using a CHEF Mapper XA system (BioRad Hercules, California). Plasmids were separated in 1.0% w/v Seakem Gold (Lonza, Bessel, Switzerland) agarose in a 0.5X TBE buffer (Invitrogen Carlsbad, CA). Size controls used were pAPEC-O1-ColBM, pAPEC-O2-ColV, and APECχ7112. Electrophoresis was carried out at 6v/cm with an angle of 120° for 20h with an initial switch time of 1s and a final switch time of 20s [20]. Following electrophoresis, the gels were stained in distilled water containing 0.5mg/ml of ethidium bromide and de-stained in water as necessary. All plasmids were visualized using UV transillumination. Individual plasmid bands were identified and excised using a clean straight razor and placed in 1.5 ml tubes. The plasmids were dissolved in Qiagen buffer QG in an incubator-shaker at 37°C for 30 minutes to remove them from the agarose. The dissolved gel fragments were subjected to sonication to fragment DNA below the maximum threshold for Qiagen column purification using a Diagenode Bioruptor Standard (Diagenode Seraing, Belgium) at high power for 30 seconds on and off time over a 5 minute period. The samples were kept on ice before, during, and post sonication. Post sonication, DNA samples were purified as per the Qiagen protocol for PCR purification using manufacturer’s specifications and eluted from purification columns using 11ul of Qiagen buffer EB. After purification, plasmids were quantified using the Q-bit 2.0 Fluorometer using the q-bit HS assay kit (Life Technologies Carlsbad, CA) according to manufacturer’s specifications.

### Plasmid Sequencing

Paired-end plasmid DNA samples were prepared for sequencing by the Dana Farber/Harvard Cancer Center DNA Resource Core using the Nextera DNA sample preparation kit (Illumina San Diego, CA) according to manufacturer’s specifications for indexed multiplexing using 50ng of input DNA. Input DNA was fragmented and tagged with the transposon, adding adapter sequences and allowing for amplification. Tagmented DNA was cleaned to remove any leftover transposon fragments. Purified DNA was then PCR amplified for 8 cycles and purified of enzymes and DNTPs. Library adapter ligation and amplification were tested by qPCR, and libraries were pooled. Library fragments in the size range of 500 to 600bp were selected using a Pippin Prep from Sage Science (Beverly, MA) according to manufacturer’s specifications. Sequencing of the tagged plasmids was carried out using an Illumina Mi-Seq machine (Illumina San Diego, CA) using a single lane. Output files ranged in size from 1 to 5 GB and were quality trimmed based on the FASTq PHRED quality scores, cutting off at Q25 and imported into the assembly software. Velvet Optimizer 2.1.4 was used to determine optimal k-mer length and coverage cutoff. Plasmid assembly was achieved using Velvet short read assembler (version 1.2.03; European Bioinformatics Institute) for final assembly [21]. In general, most plasmids assembled into one to three large contigs with few small contigs or contaminating sequences. Plasmid sequences requiring closure were closed using the previously sequenced pS88 [22] as a backbone against which to assemble small contigs left over from the Velvet assembly.

Plasmid annotation was carried out for NMEC strains sequenced in this study and elsewhere using the Prokka automated annotation analysis pipeline in order to ensure consistency in our data. The plasmids were annotated using the “-usegenus and–strain” options with a custom database generated from all APEC and NMEC plasmids in the NCBI database.

### Plasmid Core and Accessory Genome Analysis

The plasmid genomes of the fully sequenced NMEC plasmids from this study and those available online (11) were entered into an NCBI database, where an all-versus-all blast was performed to ascertain orthologous gene clusters (COGs). Genes showing >90% sequence identity, e-values of lower that 1e-10, and overlap percentages of >90% were determined to be orthologous. Orthologous genes were then sorted into strongly connected components using the algorithm of Tarjan in Perl [23]. Strongly connected components where an orthologous gene was present in each plasmid were grouped as members of a core gene set, while genes present in at least one genome were said to be members of the accessory genome.

### Plasmid Phylogenetic Analysis

Phylogenetic analysis was performed for the core genome of the NMEC and two APEC plasmids in order to compare different plasmids from different ExPEC sub-pathotypes. Core genome nucleotide cDNA sequences were extracted from files, and concatenated to form a mega alignment using custom Perl scripts. ClustalOmega [24] was used to align the nucleotide sequences and using default nucleotide settings and interleaved PHYLIP output. The PHYLIP alignment was then used in seqboot from the PHYLIP software package to create 100 bootstrapped replacements in the tree, using the–m weights option. This set of 100 weights was then used in the dnaml program from the PHYLIP software package to create a bootstrapped maximum likelihood tree using the parameters T = 2.5, S, m,1. The bootstrapped trees were then condensed using the consense program from the PHYLIP software package to give a consensus tree with bootstrap values using default parameters. This tree was then reanalyzed in dnaml using the options–u,–t 2.5, to redraw branch lengths assuming the phylogeny was represented by the bootstrap inferred tree.

### Plasmid sequencing data accession numbers

Plasmid sequences for and were deposited in GenBank under accession numbers SAMN03580091 (pNMEC-14-ColV), SAMN03580092 (pNMEC-15-ColV), SAMN03580093 (pNMEC-16-ColV), SAMN03580094 (pNMEC-19-ColV), SAMN03580095 (pNMEC-26-ColV), SAMN03580096 (pNMEC 36-ColV), SAMN03580097 (pNMEC 38-ColV), SAMN03580098 (pNMEC-49), and SAMN03580099 (pNMEC-58-ColV).

## Results

### Strain Selection, Purification and Sequence Assembly

A total of 11 plasmids was sequenced from nine NMEC strains, each isolated from different patients. Ten of eleven plasmid-containing strains were of the phylogroup B2, and the dominant serogroup was O18:K1 (Table 1). The source strains of these plasmids were previously assigned to clusters as described elsewhere [13] and were chosen for this study based on the diversity of the host strain serogroup, phylogroup, different virulence gene combinations, and cluster analysis.

Multiple plasmids were recovered from some of the NMEC strains: NMEC 15 and NMEC 36 both carried smaller cryptic plasmids. NMEC 15 contained a 114,753bp cryptic plasmid containing hypothetical and phage genes, while NMEC 36 carried a smaller 41,394bp plasmid. These plasmids contained a large number of hypothetical genes, as well as phage-related genes. Since we wished to focus our study on the plasmids in NMEC that serve a role in virulence and that are considered to be one of the defining traits of the NMEC subpathotype, all plasmids to be sequenced had to contain at least two plasmid-borne virulence genes from a list of nine commonly associated with large ExPEC virulence plasmids (*ompTp*, *hlyF*, *cvaC*, *etsA*, *iutA*, *iroN*, *tsh*, *iss*, *sitC*). Imposition of this selection criterion ensured that only plasmids containing some virulence associated genes would be included, and that cryptic or other plasmids were excluded.

Nine of the 11 plasmids sequenced in this study passed quality control and selection criteria following annotation. Two plasmids from strains NMEC 49 and NMEC 84 had a lower depth of coverage, and contained segments of contaminating DNA, resulting in poor assembly and their subsequent exclusion from analysis [Data not shown]. The plasmids pNM15c and pNM36c both contained fewer than three of the virulence associated genes. Further inspection of these plasmids showed that the majority of the plasmid genome consisted of hypothetical genes, phage genes, and genes of unknown function, and included no known virulence genes, and thus were also excluded from the analysis [Data not shown]. The resulting nine sequenced plasmids were combined with publically available NMEC plasmid sequences meeting the defined criteria described above and included the plasmids pS88 and pS286.

### ExPEC-like NMEC Virulence Plasmid Accessory Genome

**Fig 1:**
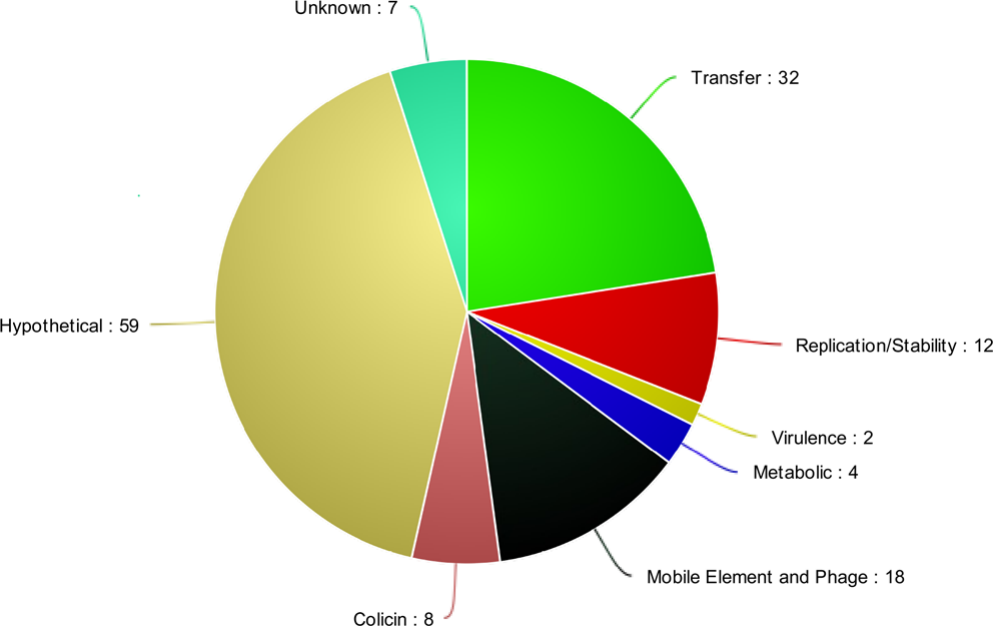
ExPEC-like Plasmid Accessory Genome Functions. Genes of the ExPEC-like NMEC plasmid accessory genome organized into functional categories based on protein prediction categories.

The virulence plasmid “accessory genome” of these 11 NMEC plasmids is defined as any COGs or single genes not found in the core genome, but present in at least one plasmid. COGs from the accessory genome identified 123 putative genes present in at least two genomes, and 197 putative genes unique to only one plasmid. Each gene’s description was derived from the consensus of the genes’ annotations. The accessory genome consisted of 142 genes appearing in at least two plasmids and 92 non-orthologous genes, appearing in only one plasmid (Fig 1). A heat map of the 142 genes present in two or more genomes is found in S1 Fig.

### ExPEC-like NMEC virulence Plasmid Core Genome

**Fig 2:**
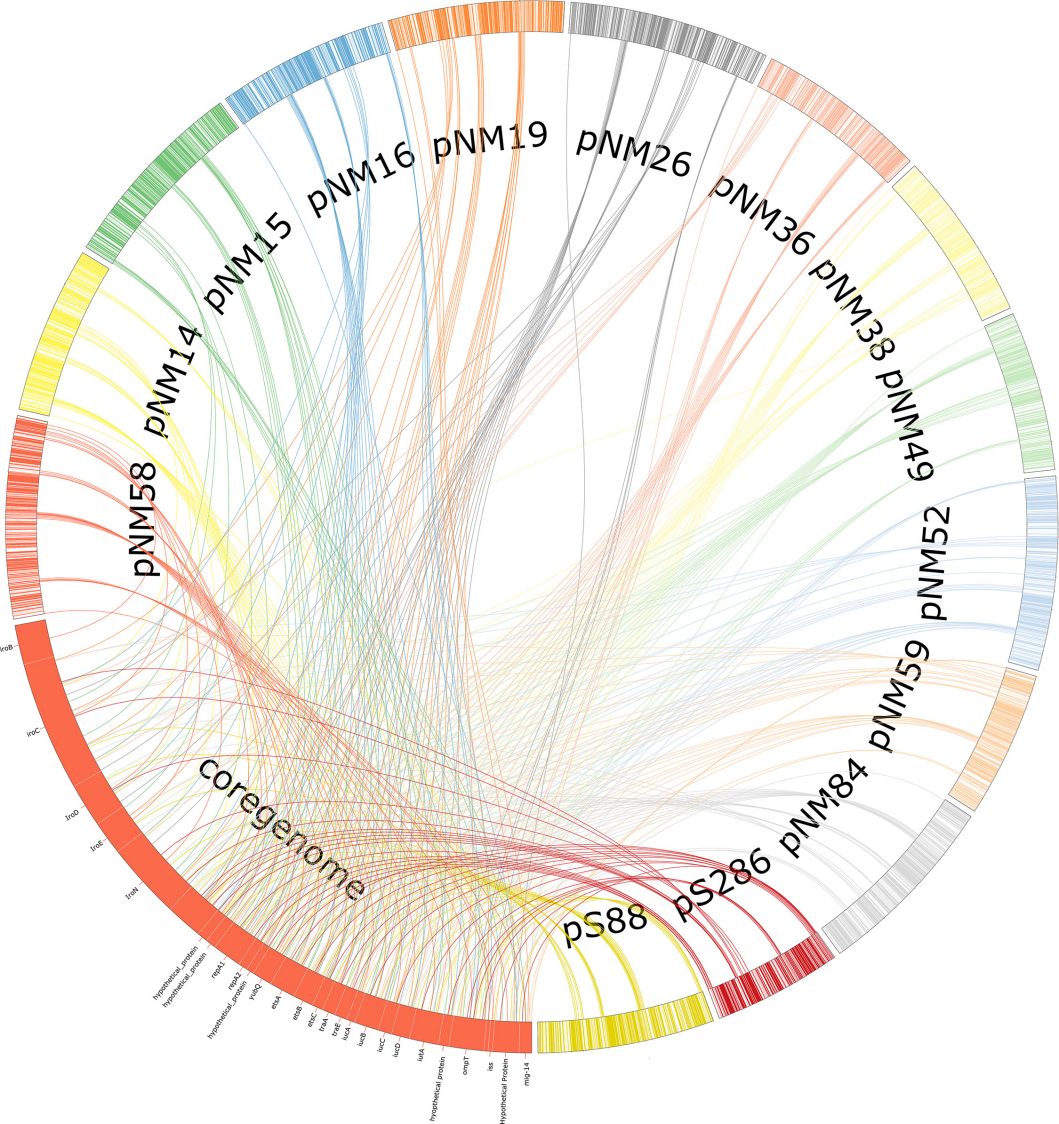
Core Plasmid Phylogeny. Visualization of ExPEC-like NMEC plasmid core genome sites. Genes within the plasmid core genome are listed in the orange core genome section, while linear representations of the chromosomes of the NMEC plasmids are arranged in the circle. Lines are drawn to the position of the core plasmid genes in their location within the plasmid to show core gene clustering

To ascertain the range of extant genes in the plasmids of NMEC strains, all sequenced and annotated NMEC virulence plasmids passing selection criteria and two previously sequenced plasmids pS88 [22] and pS286 [25] were concatenated into a cDNA BLAST [26] database. An “all versus all” blast analysis was performed to assign genes to clusters of orthologous groups (COGs). COGs in which an ortholog from each plasmid was found were considered to be “core plasmid” genes and were further subjected to protein function analysis using the CombFunc pipeline [18]. Genes in the core genome were then categorized into eight functional categories based on known function or results from the combFunc pipeline (Table 2). A preliminary core genome of 48 genes was predicted but was reduced to 38 genes by removal of insertion sequences, transposases, and phage genes (Fig 2). The core genome genes can be divided into five categories by putative functions: virulence associated; metabolic and signaling; unknown; plasmid stability and transfer; and mobile elements (Table 2).

**Table 2:** Core genes of ExPEC-like plasmids in NMEC. Core plasmid genome of sequenced plasmids and their putative functions.

Gene Name	Description	Function
*hlyF*	Hemolysin	**Virulence**
*ompT*	Outer membrane protease	
*Bor*	bacteriophage lambda bor protein	
*iroB*	putative glucosyltransferase	
*iroC*	ATP binding cassette ABC transport homolog	
*iroD*	putative ferric enterochelin esterase	
*iroE*	putative hydrolase	
*iron*	outer membrane receptor fepA	
*iucA*	aerobactin siderophore biosynthesis protein	
*iucB*	N(6)-hydroxylysine acetylase	
*iucC*	aerobactin siderophore biosynthesis protein	
*iucD*	L-lysine 6 monooxigenase	
*iutA*	ferric aerobactin receptor precursor	
*sitA*	iron/manganese transport protein, periplasmic-binding protein	
*sitB*	iron/manganese transport protein ATP-binding component	
*sitC*	Iron/manganese transport inner membrane component	
*sitD*	iron/manganese transport protein, inner membrane component	
*putative cobalamin synthase*	Putative Cobalamin synthase	**Metabolic and Signaling**
*ssbL*	DHAP synthase for shikimate pathway	
truncated enolase	putative enolase fragment	
*crcB*	supercoiling and camphor resistance protein	**Plasmid Stability and Transfer**
*repA*	RepFIB replication protein	
*repA1*	Plasmid replication protein	
*repA2*	Plasmid replication Protein	
*traA*	conjugal transfer pilin subunit	
*traE*	conjugal transfer pilus assembly protein	
*traL*	conjugal transfer pilus assembly protein	
*yacB*	putative plasmid stabalization system protein	
*finO*	conjugal transfer fertility inhibition protein	
*hypothetical mobile element*	putative mobile element protein/integrase	**Mobile Elements**
*hypothetical phage protein*	hypothetical phage protein	
*hypothetical transposase*	hypothetical transposase	
*orfA*	transposase from plasmid origin	
*hypothetical protein*	DNA Binding Function	
*hypothetical protein*	DNA polymerase	
*hypothetical protein*	Helicase activity	
*Int*	Hypothetical integrase protein	
*etsA*	putative type I secretion membrane-fusion protein	**Unknown**
*etsB*	putative type I secretion ATP binding protein	
*etsC*	putative type I secretion outer membrane protein	
*shiF*	putative MFS family membrane transport protein	
hypothetical protein	Unknown protein	
hypothetical protein	Unknown protein	
*yubQ*	putative transglycolylase	
hypothetical siderophore homolog	Putative Siderophore	
*mig-14*	putative Mig-14 protein	

The virulence-associated genes of the NMEC plasmid core included all genes of the aerobactin (*iutA/iucABCD*), *sit* (*sitABCD*), and salmochelin (*iroBCDEN)* operons. All three of these operons encode high-affinity iron-transport systems that are used by bacteria to obtain iron in low iron conditions such as those they encounter in host fluids and tissues. These operons have previously been reported in virulence plasmids of uropathogenic *Escherichia coli* (UPEC), APEC, and NMEC with high frequency and have been associated with ExPEC virulence. Another gene found in the core genome of NMEC large virulence plasmids is *iss*. This gene encodes a protein linked with increased serum survival in human *E*. *coli* isolates. Numerous studies have documented its strong alignment with virulent but not avirulent *E*. *coli* strains [27]. In addition, the genes *ompT* and *hlyF* are also found in the core genome of APEC’s large virulence plasmids. *ompT* is predicted to encode a 42-kDa pro-protein, which is processed in the membrane to a 40k-Da mature form. Mature Iss functions as a narrowly specific outer membrane endoprotease [26] that 1) cleaves paired basic residues and is involved in membrane protein turnover, 2) can degrade interferon-gamma *in-vitro* [28], and 3) cleaves the human defensin LL-37 [29]. The *hlyF* gene is predicted to encode a putative hemolysin gene; however, the exact function of this gene is unknown.

Metabolic genes of the NMEC plasmid core genome consists of three genes, *ssbL*, *eno* and a cobalamin synthesis gene. *ssbL* is a truncated enolase gene and a putative cobalamin (coenzyme B12) synthase gene. *ssbL* encodes a phospho-2-dehydro-3-deoxyheptonate aldolase or DHAP synthase. It is the first enzyme in a series of metabolic reactions known as the shikimate pathway, leading to the production of chorismate, which is responsible for the biosynthesis of the amino acids phenylalanine, tyrosine, and tryptophan and catecholate/phenolate siderophores. This gene co-locates with the *iro* operon in ExPEC plasmids [30]. The putative enolase gene found in the plasmid core genome is a very small, comprising 33% of the *eno* gene from S88 at the C-terminus at 51% identity. The *eno* gene catalyzes conversion of 2-phosphoglycerate to phosphoenolpyruvate (PEP) [31]. PEP is another reactant in the shikimate pathway for aromatic amino acid synthesis [32]. Finally, a putative cobalamin synthesis gene was also found. This putative protein contains two domains homologous to cobalamin synthesis protein p47 metal binding motifs which may act as chaperones in metal binding. Previous studies of APEC virulence have shown several vitamin synthesis pathways are crucial for APEC’s *in vivo* survival [33].

Nine genes comprise the third major gene group of the NMEC plasmid core genome. These genes are responsible for plasmid transfer and stability and include replication genes (*repA*, *repA1 and repA2*), as well as *finO*, encoding an anti-sense RNA molecule inhibiting *traJ* [34] and the supercoiling and camphor-resistance gene *crcB*. The replication genes are responsible for control of plasmid copy number in cells and prevent replication by binding to plasmid sites, thereby inhibiting DNA replication. This inhibition can be reversed by high *dnaG* expression [35–37]. Notably absent from this list are many of the *tra* genes from the type IV secretion system that responsible for plasmid conjugal transfer. They do not show up in the core because they are not present in the plasmid pS286. However, a case can be made for their inclusion since they occur in all others suggesting that pS286 is atypical, incomplete or aberrant. Blast sequence comparison between *crcB* found in this study and all other *E*. *coli* found that the *crcB* genes of NMEC plasmids and UPEC strains UCI57, UCI58, KTE76, KTE102, KTE240, and *E*. *coli* 908585 are distinct from the *crcB* gene found in other *E*. *coli*, showing only 53% amino acid identity. Such differences suggest the possibilities of different binding ligands or function. Upon initial discovery, CrcB was indicated in plasmid supercoiling and resistance to camphor when overexpressed [38]. More recent studies show CrcB to be a putative membrane protein containing a metabolite-binding RNA structure, indicated to be fluoride in these studies, which can alter downstream gene expression [39].

The NMEC plasmid core also includes mobile genetic elements and phage insertions. Mobile element genes consists of phage genes, as well as transposable elements and integration proteins. These elements recognize specific DNA sequences in the plasmid, and can cause DNA transposition for incorporating virulence genes into pathogenicity islands.

The NMEC plasmid core also includes genes of unknown function. The *etsABC* operon encodes a putative ABC transport system. It is predicted that, *etsA* encodes a putative membrane fusion protein, *etsB* encodes ATP-binding/permease protein, and *etsC* encodes an efflux protein. These genes are strongly associated with NMEC pathogenicity islands [13]. The *shiF* gene is a putative member of the COG0477 permease family. The *shiF* gene homolog lies upstream of aerobactin synthesis genes; no evidence that its function is related to this aerobactin system has been found in *E*. *coli*. The gene *yubQ* encodes an X-polypeptide. It is a protein homologous to lytic transglycosylases (LT) of family four, thought to originate from phage [40]. This class of proteins in general is thought to act on the cell wall, degrading peptidoglycan into metabolites which can be recycled in the cytoplasm. Most *E*. *coli* contain approximately seven distinct LT encoding proteins with unknown functional differences. LT proteins are essential genes in *E*. *coli*, deletion of all LT genes results in a lethal phenotype. There are two hypothetical genes of unknown function that encode no known protein domains and the combFunc pipeline was unable to predict any functions of these proteins

### Phylogenetic Analysis

**Fig 3:**
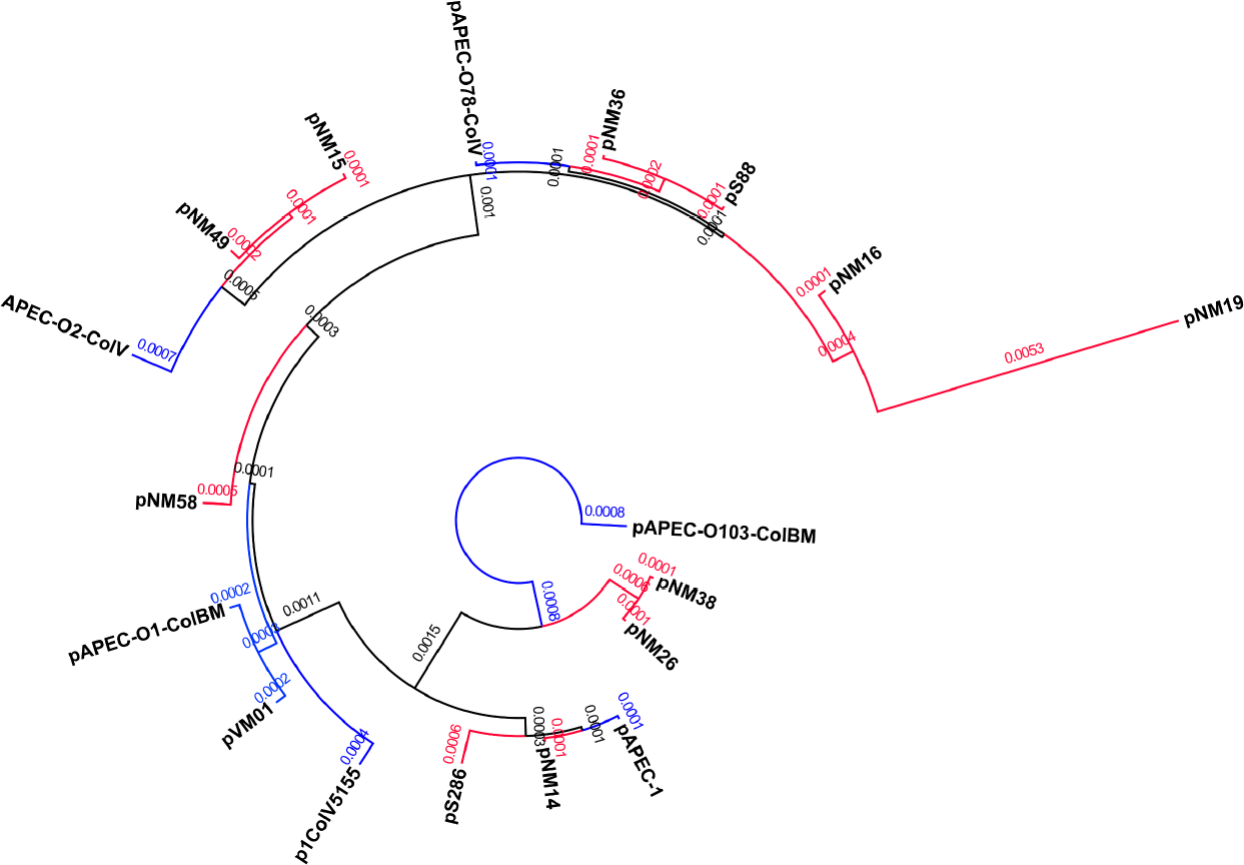
ExPEC-like plasmid phylogeny. Phylogeny of core plasmid genes of ExPEC-like NMEC virulence plasmids and APEC plasmid genes. Alignment was based on concatenation of core genes shared between APEC and NMEC plasmids. Genes were identified using an all-versus-all nucleotide blast using the blastn algorithm using default parameters. Blast matches sorted into strongly connected components using Tarjan’s algorithm. Nucleotide sequences from strongly connected groups were extracted in a randomized order and concatenated. Bootstrap analysis was performed using Phylip seqboot algorithm creating 10 sets of 10 weights using default parameters, which were then used in 10 separate runs of dnaml using the parameters T 2.5, S, M, 1. Phylip consense was used to condense all output trees into a consensus bootstrap analysis using default parameters

The NMEC core plasmid genome was used to develop an ExPEC plasmid phylogeny. To relate the evolution of these plasmids to other *E*. *coli* plasmids, three APEC plasmids, pAPEC-O1-ColBM, pAPEC-O2-ColV and pAPEC-O103 were included in the analysis. These plasmids meet the same three criteria for inclusion as described above and were included in the analysis to better understand differences between plasmids from different ExPEC subpathotypes. However, UPEC plasmids were not included in this analysis as no publically available sequenced UPEC plasmids met inclusion criteria. The APEC/NMEC core plasmid genome consisted of 15 genes (21,496bp), containing the *iro* and *iut* operons as well as replication proteins *repA* and *repB*, which was used to generate a plasmid phylogeny (Fig 3). Additionally, one outlier was observed in this analysis: pAPEC-O103, which is a hybrid plasmid containing a pathogenicity island and multi-drug resistance island and lacking the complete *tra* operon. As a result of this analysis, it appears that there are at least two lineages of NMEC virulence plasmids. In addition, as a result of the comparative analysis, it appears that APEC and NMEC plasmids cannot be differentiated solely based on the presence or absence of genes in a core genome of plasmid.

## Discussion

The contributions made to ExPEC pathogenesis by large virulence plasmids has received increased scrutiny in recent years. In part, this scrutiny is due to creation of the reagents needed for their study, such as plasmid genomic sequences [7, 11, 22, 30], and dissemination of new plasmid isolation protocols that enabled harvesting of large, naturally occurring plasmids from wild-type bacterial strains [41]. Previous to that the presence of large ExPEC plasmids was likely overlooked [8, 16]. Here, in an effort to define the core and accessory genome of NMEC plasmids, nine new plasmids isolated from NMEC were sequenced. Clusters 5 and 9 contained only a single isolate, while clusters 2 and 3 primarily consisted of human fecal *E*. *coli* biasing the strain selection towards groups 6–8, which were determined to be the most pathogenic and most common clusters for NMEC in the analysis [13]. Efforts were made to sequence strains from different serogroups where multiple options existed, attempting to capture the dominant O-types of O18, O83, and O7, while still sequencing representatives from less common serogroups, such as O14, O21, as well as untypeable NMEC. Selecting for strains outside of the predominant B2 phylogroup is difficult, as our collection of 94 NMEC isolates contains are predominantly B2 strains (78%), with the next most common phylogenetic group being F, representing 6.8% of the isolates in the full 91 sample collection. Finally, K type was used if all other parameters from the analysis were the same, which included one strain of the K80 type in the analysis, as well as multiple untypeable capsules. The previously published NMEC plasmids pRS218 and pCE10 were excluded from the analysis due to the absence of all virulence genes used to define a virulence plasmid in this study. Further analysis of the chromosomes of strain CE10 contained the *iutABCD* and *sitABCDC* genes present in the chromosome, while RS218 did not possess any of these plasmid virulence genes on the plasmid or the chromosome.

Assembled plasmids varied largely in terms of size, but surprisingly had similar GC content and numbers of open reading frames, indicating that a large proportion of these plasmids may not encode functional proteins. This seems to be borne out by the significant number of hypothetical genes and pseudo genes in the accessory genome of the NMEC plasmids. It is unclear whether this result suggests that some plasmids are accumulating genetic information that is not used, or whether they have not yet shed excess genetic information that may not be functional or whether these hypothetical or pseudo genes have an unknown function. Genes related to antibiotic resistance were not found in any of these plasmids with only one plasmid harboring a single putative antibiotic resistance gene, distinct from the APEC hybrid plasmid pAPECO103 encoding both resistance and virulence genes [42]. The lack of resistance genes is corroborated by the lack of multiple drug resistant strains in our NMEC collection [43], as well as those available publically for other NMEC strains. Furthermore a complete and intact transfer region was not found on all of the NMEC plasmids, suggesting some loss of the transmissible capability during the evolution course.

The core genome of the NMEC plasmids contains 47 genes, most of which encode components of iron uptake systems. Iron uptake systems are necessary for the full virulence of septicemia associated *E*. *coli* [7, 13, 22], uropathogenic *E. coli [44]*, and avian pathogenic *E*. *coli* [14, 45–47]. Although it has not yet been reported, this study suggests that iron acquisition is important for NMEC virulence since human serum and cerebrospinal fluid (CSF) are also iron-deficient environments. Indeed, the presence of iron acquisition genes in NMEC isolates has been well established [7, 13] and NMEC, UPEC, and APEC share a similar content of iron uptake systems that are not found in non-virulent fecal *E*. *coli* strains from both human and animal sources [13]. Moreover, unpublished data from our group has found that the expression of several iron uptake systems was significantly upregulated when NMEC were cultured in human serum and CSF compared to strains cultured in LB (unpublished data). The data presented here indicate that three iron uptake systems the ferric aerobactin system (*iutA/iucABCD*), the salmochelin siderophore system (*iroBCDEN)*, and SitABCD system, have been associated with ExPEC virulence, and are encoded by core the genome of NMEC large virulence plasmids. Interestingly, the metabolism gene (*ssbL*) found in the core genome of NMEC virulence plasmids appears to be co-located with these iron genes. This *ssbL* gene encodes a DHAP synthase which may boost production via the shikimate pathway of aromatic amino acids and are necessary for large amount production of enterobactin, salmochelin SX, and yersiniabactin [30]. The finding of *ssbL* gene in the core genome of the NMEC virulence plasmid appears to reinforce the importance of the iron uptake system for NMEC virulence.

Based on the findings published thus far, NMEC’s ability to cause meningitis requires at least two major steps and one of them is intravascular multiplication leading to high-level bacteremia. Thus, NMEC must be able to resist or avoid the detrimental impact of the host complement, antimicrobial peptides, polymorphonuclear leukocytes, and other specific and innate immunity factors. In the core genome of NMEC virulence plasmids, the *iss* gene was found, which is homologous to the *bor* gene of bacteriophage λ. The Iss protein increases *E*. *coli*’s serum survival likely by restricting C3 deposition on the bacterial surface [27, 48] and blockage of the membrane attack complex [49]. Another core genome gene of the NMEC virulence plasmid involved in resistance to innate immunity is *ompT*. The *ompT* gene encodes an outer membrane protease [26] that can degrade interferon-gamma. More recent studies on *ompT* in enteropathogenic *E*. *coli* (EPEC) and enterohaemorrhagic *E*. *coli* (EHEC) showed *ompT* cleaves the human defensin LL-37 [29], a pore forming antimicrobial peptide expressed by neutrophils, epithelial cells, and bone marrow that serves both antimicrobial and immunomodulatory functions. The *ompT* gene in the core genome of NMEC virulence plasmids shares approximately 74% of amino acid identity to those encoded by EPEC and EHEC. Thus, it could be inferred that OmpT can degrade interferon and human antimicrobial peptide increasing NMEC’s resistance to human innate immunity, thus contributing to NMEC’s pathogenesis. In addition, several other accessory genome genes of NMEC virulence plasmids were found to contribute to NMEC’s resistance to host immunity. The gene *tsh*, which has been previously reported to be an important virulence factor for closely related APEC strains and is present in 11 to 50 percent of NMEC samples, encodes a protein able to cleave leukocyte glycoproteins, impair chemotaxis and transmigration, and activate leukocytes’ programmed cell death [50].

The homolog of CrcB was shown to be involved in the chromosome condensation and plasmid supercoiling, causing a secondary effect of resistance to camphor, which decreases chromosome condensation [38]. Overexpression of the gene increases chromosomal condensation and can correct nucleoid morphology defects in some mutants, and may help these large plasmids partition into daughter cells. As we know the *E*. *coli* chromosome, like its eukaryotic counterparts, must be condensed approximately 2000-fold to fit inside an *E*. *coli* cell. Given the large size of the virulence plasmids for NMEC, it is likely that the *crcB* gene is carried on the plasmid is necessary to fit the plasmid into the cells, and may increase the likelihood of the plasmid successfully passing to daughter cells post replication.

In conclusion, this is the first study to define the core and accessory genome of ExPEC-like NMEC plasmid, and perform a phylogenetic analysis. The role of the core genome of NMEC plasmids is primarily to increase the bacteria’s *in-vivo* fitness by enhanced uptake of iron in a deficient environment, and resistance to host innate immunity as well as maintain the plasmid in the bacterial population with systems for conjugation and plasmid stability. Phylogenetic analysis of the NMEC plasmids shows that at least two lineages can be discerned and they are nearly indistinguishable from those of the APEC plasmid. This finding supports previous research inferring close genetic relationships between APEC and NMEC plasmids and overlap in a large number of pathogenicity islands [9, 51, 52]. We hope this and future studies lead to a better understanding of the molecular mechanisms by which NMEC large plasmids contribute to virulence and their evolution.

## Supporting Information

S1 Fig Heat map of the ExPEC-like NMEC accessory genome. Strains containing listed genes are shown in red, absent genes are shown in black. The plasmid accessory genome contained a large number of hypothetical genes, genes of unknown function, phage genes, and insertion sequences. Other notable genes in the accessory genome included *tsh* the temperature sensitive hemagglutinin, and a homologue of the outer membrane protease *ompT*, though all plasmids carried another distinct copy of the *ompT* protease.
(PDF)
