# Supplementary material for: Genetic Characterization of ExPEC-Like Virulence Plasmids among a Subset of NMEC
Source: PLoS One. 2016 Jan 22;11(1):e0147757. doi: 10.1371/journal.pone.0147757 (PMC4723317; doi:10.1371/journal.pone.0147757)
Supplement: S1 Fig — Strains containing listed genes are shown in red, absent genes are shown in black. The plasmid accessory genome contained a large number of hypothetical genes, genes of unknown function, phage genes, and insertion sequences. Other notable genes in the accessory genome included tsh the temperature sensitive hemagglutinin, and a homologue of the outer membrane protease ompT, though all plasmids carried another distinct copy of the ompT protease. (PDF) [file pone.0147757.s001.pdf]

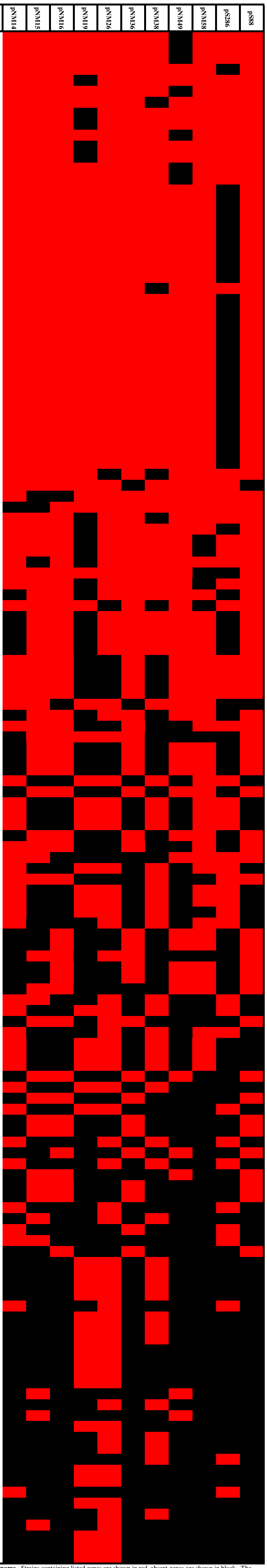

Gene Name	% incidence
cvaA	91%
cvaB	91%
cvaC	91%
hypothetical protein	91%
hypothetical protein	91%
hypothetical protein	91%
hypothetical protein	91%
hypothetical protein	91%
hypothetical protein	91%
PQ loop repeat protein	91%
psiA	91%
psiB	91%
putative dehydrogenase	91%
putative dehydrogenase	91%
traB	91%
traC	91%
traD	91%
traF	91%
traG	91%
traH	91%
traI	91%
traK	91%
traN	91%
transposase IS605 family, IS200 group	91%
traP	91%
traQ	91%
traR	91%
traT	91%
traU	91%
traV	91%
traW	91%
traX	91%
trbA	91%
trbB	91%
trbC	91%
trbD	91%
trbE	91%
trbG	91%
trbI	91%
trbJ	91%
hypothetical protein	82%
hypothetical protein	82%
hypothetical protein	82%
hypothetical protein	82%
hypothetical protein	82%
ssb	82%
traJ	82%
traM	82%
transposase subunit	82%
traS	82%
traY	82%
adenine-specific DNA methylase	73%
hypothetical protein	73%
hypothetical protein	73%
hypothetical protein	73%
hypothetical protein	73%
hypothetical protein	73%
insL	73%
IS2 transposase	73%
sopA	73%
sopB	73%
hypothetical protein	64%
hypothetical protein	64%
hypothetical protein	64%
cia	55%
flmC	55%
hypothetical protein	55%
hypothetical protein	55%
hypothetical protein	55%
hypothetical protein	55%
hypothetical protein	55%
hypothetical protein	55%
hypothetical protein	55%
hypothetical protein	55%
hypothetical protein	55%
insB	55%
insI	55%
ompT homolog	55%
srnB	55%
tsh	55%
def	45%
helix-turn-helix protein	45%
hypothetical protein	45%
hypothetical protein	45%
hypothetical protein	45%
hypothetical protein	45%
hypothetical protein	45%
hypothetical protein	45%
hypothetical protein	45%
hypothetical protein	45%
imm	45%
transposase ORF A, IS3 family	45%
TrbJ	45%
vagD	45%
vapB	45%
yigB	45%
artA	36%
hypothetical protein	36%
hypothetical protein	36%
hypothetical protein	36%
hypothetical protein	36%
hypothetical protein	36%
hypothetical protein	36%
insF	36%
yacA	36%
yacB	36%
yacC	36%
hypothetical protein	27%
hypothetical protein	27%
hypothetical protein	27%
hypothetical protein	27%
hypothetical protein	27%
parM	27%
Plasmid stability protein	27%
ppaC	27%
resolvase	27%
sok	27%
Tn3 transposase DDE domain protein	27%
Tn3 transposase DDE domain protein	27%
uspC	27%
cbi	18%
cma	18%
cmi	18%
CobQ/ParA nucleotide binding protein	18%
hemolysin expression modulating protein	18%
hypothetical protein	18%
hypothetical protein	18%
hypothetical protein	18%
hypothetical protein	18%
hypothetical protein	18%
hypothetical protein	18%
hypothetical protein	18%
hypothetical protein	18%
hypothetical protein	18%
hypothetical protein	18%
hypothetical protein	18%
hypothetical protein	18%
Plasmid stability protein	18%
resolvase	18%
yhdJ	18%

Fig S1. Heat map of the ExPEC-like NMEC accessory genome. Strains containing listed genes are shown in red, absent genes are shown in black. The plasmid accessory genome contained a large number of hypothetical genes, genes of unknown function, phage genes, and insertion sequences. Other notable genes in the accessory genome included tsh the temperature sensitive hemagglutinin, and a homologue of the outer membrane protease ompT, though all plasmids carried another distinct copy of the ompT protease.
